# Gut Microbiota in Tibetan Herdsmen Reflects the Degree of Urbanization

**DOI:** 10.3389/fmicb.2018.01745

**Published:** 2018-07-31

**Authors:** Huan Li, Tongtong Li, Xiangzhen Li, Guanhong Wang, Qiang Lin, Jiapeng Qu

**Affiliations:** ^1^Institute of Occupational Health and Environmental Health, School of Public Health, Lanzhou University, Lanzhou, China; ^2^Department of Applied Biology, College of Biotechnology and Bioengineering, Zhejiang University of Technology, Hangzhou, China; ^3^Key Laboratory of Environmental and Applied Microbiology, Environmental Microbiology Key Laboratory of Sichuan Province, Chengdu Institute of Biology, Chinese Academy of Sciences, Chengdu, China; ^4^The Rowland Institute at Harvard, Harvard University, Cambridge, MA, United States; ^5^Institute of Soil Biology, Czech Academy of Sciences, České Budějovice, Czechia; ^6^Key Laboratory of Adaptation and Evolution of Plateau Biota, Northwest Institute of Plateau Biology, Chinese Academy of Sciences, Xining, China; ^7^Qinghai Provincial Key Laboratory of Restoration Ecology in Cold Region, Xining, China

**Keywords:** gut microbiota, urbanization, beta diversity, network interaction, environmental filtering, lifestyle

## Abstract

Urbanization is associated with shifts in human lifestyles, thus possibly influencing the diversity, interaction and assembly of gut microbiota. However, the question regarding how human gut microbiota adapts to varying lifestyles remains elusive. To understand the relationship between gut microbiota and urbanization, we compared the diversity, interaction and assembly of gut microbial communities of herdsmen from three regions with different levels of urbanization, namely traditional herdsmen (TH), semi-urban herdsmen (SUH) and urban herdsmen (UH). The relative abundance of *Prevotella* decreased with the degree of urbanization (from TH to UH), whereas that of *Bacteroides, Faecalibacterium*, and *Blautia* showed an opposite trend. Although the alpha diversity measures (observed OTUs and phylogenetic diversity) of gut microbiota were unaffected by urbanization, the beta diversity (Jaccard or Bray–Curtis distances) was significantly influenced by urbanization. Metagenome prediction revealed that the gene functions associated with metabolism (i.e., carbohydrate and lipid metabolism) had significant differences between TH and UH. Network analysis showed that the modularity increased with the degree of urbanization, indicating a high extent of niche differentiation in UH. Meanwhile the trend of network density was opposite, indicating a more complex network in TH. Notably, the relative importance of environmental filtering that governed the community assembly increased with the degree of urbanization, which indicated that deterministic factors (e.g., low-fiber diet) play more important roles than stochastic factors (e.g., stochastic dispersal) in shaping the gut microbiota. A quantification of ecological processes showed a stronger signal of variable selection in UH than TH, implying that different selective pressures cause divergent gut community compositions due to urban lifestyles. Our results suggest that beta diversity, network interactions and ecological processes of gut microbiota may reflect the degree of urbanization, and highlight the adaptation of human gut microbiota to lifestyle changes.

## Introduction

Ecological migration has become a common strategy for environmental protection in China to cope with environmental risk and stress. It refers to the transition of human lifestyle from traditional rural to modern urban (Jia, 2016). The process of urbanization is associated with the shifts of lifestyle factors, such as diet and geography. These lifestyle factors have influenced the composition of gut microbial communities according to cross-cultural comparative microbiota analyses (Obregon-Tito et al., 2015; Gomez et al., 2016; Winglee et al., 2017). Particularly, in comparison with the traditional rural cultures, urbanization or industrialization may result in decreased microbial diversity, including loss of bacterial taxa associated with dietary fiber, and increased bacterial lineages associated with animal protein-rich or industrial food (Martinez et al., 2015; Gomez et al., 2016; Winglee et al., 2017). Urban diet and lifestyles may shape some specific gut bacterial taxa, which play important roles in high incidence of various diseases, such as obesity, diabetes, cancer and cardiovascular diseases (O’Keefe et al., 2015; Zhou et al., 2015; Carrillo-Larco et al., 2016; Song et al., 2016). Thus, comparative analysis of gut microbiota among human populations in different levels of urbanization will facilitate our understanding regarding the effects of urban diet and lifestyles on human health.

Previous studies have revealed several some potential associations between the gut microbial diversity and urban (or industrial) diet or lifestyles based on cross-cultural comparative microbiota analyses (Schnorr et al., 2014; Obregon-Tito et al., 2015). However, most of these studies focused on human subjects with different ethnicities and cultures, or people living in geographically distant regions. Only few studies have explored the relationship between gut microbial diversity and the degree of urbanization in human individuals who have similar ethnicity, culture and are in close geographical areas. In addition to microbial diversity, gut microbes may form networks through various interactions (Ramayo-Caldas et al., 2016), such as competition and mutualism. These network interactions are common and particularly important for understanding the structure of gut microbial communities, and may reveal the niche shared by community members (Faust and Raes, 2012; Kara et al., 2012). Recent studies have investigated the large and complex microbial communities, and revealed several new unseen network co-occurrence patterns, including niche specialization, strong non-random associations (Faust and Raes, 2012) and succession of deterministic processes based on network topology (Lin et al., 2017). Thus, network analysis of gut microbial communities may reveal several common system-level properties associated with specific lifestyle.

Although several reports have shed light on the composition and diversity of traditional rural and urban people (Schnorr et al., 2014; Gomez et al., 2016), relatively few studies were involved in the ecological processes that govern the gut microbiota. Ecological community assembly involved either stochastic or deterministic processes, or both (Stegen et al., 2012, 2013; Zhou et al., 2014). When a community is governed by stochastic processes, the composition and diversity are unpredictable. Stochastic events, such as stochastic dispersal, extinction, colonization and drift, may play a major role in the assembly of microbial communities (Purves and Turnbull, 2010; Rosindell et al., 2012). Controlling the community diversity by regulating external environmental factors seems impossible. In contrast, when a community is shaped by deterministic processes, the community diversity and structure are directional and predictable. Filtering or selection may result in the formation of similar communities in similar environmental conditions (Zhou et al., 2014). External environmental factors may regulate and control the community structure, diversity and directionality. Vellend’s community ecology theory assumes that community diversity is formed by four ecological processes, namely selection, dispersal, diversification and drift (Vellend, 2010). Diversification processes are relatively important at long evolutionary scales. Thus, Vellend et al. (2014) only focused on the remaining three ecological processes, that is, selection, drift and dispersal. They considered that selection belongs to deterministic processes, drift belongs to stochastic processes, and dispersal may be deterministic, stochastic or both processes, depending on the situation (Vellend et al., 2014). All the three processes may govern the assembly of the gut microbiota in some ecological systems (Yan et al., 2016). However, the ecological processes are dynamic and changeable in different systems. Previous studies mainly focused on gut microbial diversity and urbanization (Schnorr et al., 2014; Obregon-Tito et al., 2015), however, the relative contribution of the ecological processes in response to urbanization remains unclear. Addressing this issue may help us understand the community assembly mechanisms in response to host ecology. Here, we determine how the diversity, interaction and assembly of gut microbiota changes in response to recent urbanization.

China is an ideal place to investigate the relationship between urbanization and gut microbiota, because many areas (e.g., Sanjiangyuan) in this country have only began to undergo massive urbanization due to the implementation of ecological immigration policy. In the Qinghai–Tibet Plateau, a mix of traditional herdsmen (TH), semi-urban herdsmen (SUH) and urban herdsmen (UH) may be observed, indicating the large differences in major urbanization-related shifts (e.g., diet). In this study, we analyzed and compared the diversity, interaction and assembly of gut microbial communities of TH, SUH, and UH in Tibet in the Qinghai–Tibet Plateau. We addressed the following four major questions: (a) How do bacterial taxa and diversity change in response to urbanization? (b) Which gene functions show differences between traditional and urban herdsmen? (c) Which network topological characteristics of the gut microbiota reflect the degree of urbanization? (d) Is there a link between community assembly processes and degree of urbanization? Our results demonstrated that diversity, network interactions and ecological processes of the gut microbiota in Tibetan herdsmen may reflect the degree of urbanization.

## Materials and Methods

### Study Sites, Sample Collection and Ethical Standards

Fresh stool samples were collected from 24 individuals from Hainan State on the Qinghai–Tibet Plateau. Since 2004, the state started to implement the ecological immigration project. The lifestyle of the traditional Tibetan herdsmen has undergone considerable changes, and several herdsmen are experiencing a transition in lifestyle from traditional to semi-urban to urban. The samples were collected from September to October of 2016 with consent from the 24 individuals. A total of 24 samples were collected from the three adjacent areas of the Hainan State. These samples were divided into three groups, namely TH (*N* = 8), SUH (*N* = 8), and UH (*N* = 8). The samples of group UH were from Guinan County (35°35′03″ N, 100°45′03″E), whereas the sampling sites of the group TH and SUH were close to the Zeku and Tongde Counties (35°09′31″N, 100°44′10″E; 35°15′23″ N, 100°48′34″E), respectively. The geographical distance among sampling sites was approximately 30 km. No participants had used any antibiotics within the past 3 months. None was pregnant, lactating or morbid. For each participant, approximately 5 g of fresh feces were collected, and placed in 10 ml sterile tubes, and then stored in liquid nitrogen (-196°C). All samples were transferred to our laboratory within 24 h and kept at -40°C until DNA extraction.

Our work was approved and supported by the Ethics Committee of Chengdu Institute of Biology, Chinese Academy of Sciences. Written informed consents from all the participants were submitted to the ethics committee. Sample collection strictly followed the relevant guidelines.

### Sample Preparation and High-Throughput Sequencing

Genomic DNA was extracted from stool samples using the MoBio PowerSoil Kit (Mo Bio Laboratories Inc., Carlsbad, CA, United States). Detailed procedures of PCR amplification and gel extraction were performed as described previously (Li et al., 2016a,b,c). The V4–V5 region of the microbial 16S rRNA gene was PCR-amplified using the universal primer pair 515F/909R (Tamaki et al., 2011). The 5′-end of the 515F primer was tagged with 12-bp unique barcodes to split the sequences of each sample. PCR amplification was performed in duplicate for each sample to minimize bias. Finally, the purified PCR products were pooled in equal molar concentrations and sequenced using an Illumina MiSeq sequencer (Illumina, San Diego, CA, United States) with a paired-end protocol of 300 cycles (Reagent Kit V3).

### Bioinformatics Analysis

The raw sequences were analyzed and processed in the Quantitative Insights Into Microbial Ecology (QIIME v1.7.0) pipeline (Caporaso et al., 2010). The paired-end sequences were merged with FLASH software (Magoc and Salzberg, 2011). Sequences of each sample were split based on their unique barcodes. Sequence analysis followed the methods in our previous reports (Li et al., 2016a,b,c). Briefly, after filtering low-quality sequences, chimeras and chloroplasts, all the remaining sequences were then clustered into operational taxonomic units (OTUs) at a 97% sequence similarity using UCLUST algorithm (Edgar, 2010). The most abundant sequence of each OTU was selected as representative sequence and then aligned against the Greengenes 13_8 reference database (DeSantis et al., 2006) using PyNAST. Representative sequences were classified using the RDP classifier (Wang et al., 2007). After microbial taxonomies were assigned, OTUs that were affiliated with Eukaryota, Archaea, and those not classifying to bacteria were removed from our datasets. Singletons were also filtered out. The OTU table was converted and filed in BIOM format for the subsequent analysis.

In order to correct for uneven sequencing depth, each sample was normalized to 24,447 sequences. To evaluate alpha diversity indices, observed species and phylogenetic diversity were calculated for each group. In addition, the rarefaction curves based on the observed species at OTU level were calculated. To assess beta diversity, we calculated the Jaccard (for community composition) and Bray–Curtis dissimilarity metrics (for community structure) of bacterial communities (Dill-McFarland et al., 2015). The Jaccard distance is based on the presence/absence of OTUs, whereas the Bray–Curtis distance is based on the abundance of OTUs. The differences in the overall community composition and structure among all groups were visualized using the non-metric multidimensional scaling (NMDS) ordination plots of Jaccard and Bray–Curtis distance matrices.

The original 16S rRNA data were available at the European Nucleotide Archive by accession NO. PRJEB23227^1^.

### Statistical Analysis

To compare the differences of bacterial communities among lifestyles, permutational multivariate analysis of variance (PERMANOVA) (McArdle and Anderson, 2001) and analysis of similarity (ANOSIM) (Dill-McFarland et al., 2015) were used to evaluate whether gut microbiota structure was significantly different based on the Jaccard and Bray–curtis distances using the ‘adonis’ and ‘anosim’ procedure in the R ‘vegan’ package. The model also comprised some other host characteristics, including age, body weight, height and gender. Mann–Whitney U-tests were used to identify differences of alpha diversity measures among groups, and to compare intra- and inter-group dissimilarities in gut microbiota composition. The indicator value (IndVal) was employed to identify the indicative bacterial genera (mean relative abundance > 0.1%) associated with each group (Dufrêne and Legendre, 1997). Only those IndVals that were significant (*P* < 0.05) and more than 0.4 were considered using the package ‘labdsv’ in R (Roberts, 2007). *P*-values were corrected using false discovery rate (FDR).

Phylogenetic Investigation of Communities by Reconstruction of Unobserved States (PICRUSTv1.1.2) (Langille et al., 2013) was used to predict the abundance of gene functions based on our 16 S rRNA sequences. The average Nearest Sequenced Taxon Index values of all the samples (0.12 ± 0.04) suggested the comparable predictive accuracy of our communities compared with those achieved for mammalian gut microbiotas (Langille et al., 2013). The NMDS plots of the Bray–Curtis dissimilarity based on the gene functions at level 3 was calculated to investigate the overall functional differences among groups. PERMANOVA and ANOSIM were used to evaluate the differences in functional profiles among groups. Significant differences (*t*-tests, Bonferroni-corrected) of the predicted gene functions were tested between TH and UH at level 3.

### Network Analysis and Putative Keystone Taxa in Herdsmen

Phylogenetic molecular ecological networks (pMENs) were constructed using an open-accessible pipeline (Deng et al., 2012) to identify the interspecies interaction of gut microbiota in the three groups (TH, SUH, and UH). The procedure of network construction was described previously (Li et al., 2017). We used the same cutoff values (0.89) for different networks (FDR-q < 0.001). A set of topological features were calculated, including the modularity (M), average degree (avgK), average clustering coefficient (avgCC), density (D), connectedness (Con) and positive co-occurrences (Deng et al., 2012). The ecological networks were visualized using Cytoscape 3.0.2 (Saito et al., 2012).

To identify the keystone species in bacterial networks, the within-module connectivity (*Zi*), and among-module connectivity (*Pi*) were calculated based on the methods of Deng et al. (2012). Hub and connector taxa were determined by *Zi* and *Pi* values. All nodes in a network were divided into four categories, including peripherals (*Zi* < 2.5 and *Pi* < 0.62), connectors (*Pi* > 0.62), network hubs (*Zi* > 2.5 and *Pi* > 0.62), and module hubs (*Zi* > 2.5). Those nodes belonging to module hubs, network hubs and connectors were regarded as putative keystone species in the microbial community (Li et al., 2017). The keystone specie of the group TH, SUH, and UH were identified following the aforementioned rules.

### Evaluation of Ecological Processes for Community Assembly

Community assembly is governed by either deterministic or stochastic processes, or both (Stegen et al., 2012, 2013; Zhou et al., 2014). First, to understand the overall ecological processes based on taxonomy-based distance metrics, a null model analysis was performed to reveal whether the observed beta diversity values (Jaccard and Bray–Curtis distances) were significantly different from the null expectation based on permutational analysis of multivariate dispersion (PERMDISP) (Werner and Nicholas, 2010; Zhou et al., 2014). If the observed beta diversity was significantly distinct from the null model, then deterministic processes play a major role in the community assembly. Conversely, if the observed beta diversity was not significantly different from the random null model, then stochastic processes play a more important role in the community assembly. In addition, Nearest Taxon Index (NTI) and Mean-nearest-taxon-distance (MNTD) were used to qualitatively evaluate the deterministic or stochastic processes of community assembly (Meyerhof et al., 2016; Yan et al., 2016) based on phylogenetic diversity. These phylogenetic analyses were performed using the ‘picante’ package (Kembel et al., 2010) and employing the ses.mntd command with null model = ‘taxa. labels’ and abundance.weighted = TRUE with 1000 randomizations. The ses.mntd command simulates a null distribution of MNTD values and calculates the difference between the observed MNTD and the mean expected MNTD divided by the standard deviation of the expected values. NTI is the negative of the output of ses.mntd and quantifies the number of standard deviations that the observed MNTD is from the mean of the null distribution. If mean NTI values of bacterial communities for each group were significantly greater than 0, then we conclude that community assembly is mainly governed by phylogenetic clustering. If mean NTI values for each group were significantly less than 0, then phylogenetic overdispersion may play a more important role in structuring the microbial community (Stegen et al., 2012). Further, a larger absolute magnitude of NTI value reflects the stronger effects of deterministic processes (Yan et al., 2016).

To determine which ecological processes (variable selection, homogeneous selection, dispersal limitation, homogenizing dispersal or drift) may govern bacterial community assembly of each group, the methods of Stegen et al. (2013) were used in calculating the potential ecological processes. Briefly, the phylogenetic diversity of bacterial communities between a pair of samples was calculated. R package was applied to quantify the weighted beta nearest taxon index (β-NTI). The combination of β-NTI and Bray–Curtis-based Raup-Crick (RC_bray_) was further used to speculate the relative importance of the major ecological processes that govern the bacterial communities. The values of β-NTI > 2 or <-2 represented the community turnover determined by the variable or homogeneous selection. If -2 < β-NTI < 2 and RC_bray_ > 0.95 or <-0.95, then the community turnover was determined by dispersal limitation or homogenizing dispersal. If 2 < β-NTI < 2 and -0.95 < RC_bray_ < 0.95, then the community turnover was drift (referred to as “undominated” processes) (Stegen et al., 2015).

## Results

### Sample Characteristics and Dietary Information

The age, height, weight, and gender of herdsmen were identified (**Table 1**). Age, height, and weight showed no significant differences among different groups (one-way ANOVA, *P* > 0.05). However, the dietary configurations underwent remarkable shifts from TH to SUH to UH. For example, the main food of TH was Zanba (∼50% high-fiber food), while the main diet composition of UH was noodles and rice, which has low fiber content compared with Zanba (**Table 1**). In compared with TH, UH had a more diverse food spectrum, including various types of meat, vegetable and fruit (**Table 1**).

**Table 1:** Characteristics of the Tibetan herdsmen studied.

	Traditional herdsmen (TH, *n* = 8)	Semi-urban herdsmen (SUH, *n* = 8)	Urban herdsmen (UH, *n* = 8)
Age (years ± SD)	39 ± 16	37 ± 17	35 ± 14
Height	166 ± 8	165 ± 7	175 ± 6
Weight	69.5 ± 15.2	65.3 ± 25	69 ± 11.6
Gender (male/female)	5/3	4/4	4/4
Main food	Zanba	Zanba, noodle, rice	Noodles, rice
Animal food	Beef, mutton	Beef, mutton, eggs (Occasional)	Beef, mutton, pork, eggs (often)
Vegetables	Potato, cabbage	Potato, cabbage, radish, lettuce	Various vegetables
Frequency of eat fruits	Seldom	Occasional	Everyday
Main fruits	Few	Apple, banana	Various fruits

### Changes of Bacterial Taxa With Urbanization

**FIGURE 1:**
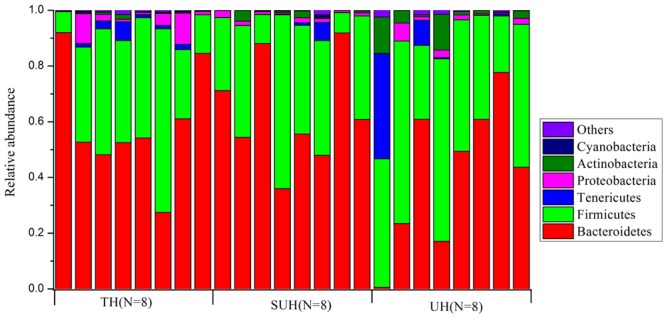
The composition of gut bacterial communities at phylum level in traditional herdsmen (TH), semi-urban herdsmen (SUH) and urban herdsmen (UH). Only those phyla with mean relative abundance > 0.1% across all samples were shown.

Gut microbiota compositions were profiled through MiSeq sequencing of 16S rRNA gene from 24 samples from the three groups (TH, SUH, and UH). A total of 1,149,039 raw reads were obtained. After removing the low-quality sequences, chimeras, chloroplasts, and those that were not classified as bacteria, we obtained 1,088,190 sequences, with an average number of 45,341 sequences per sample (range 24,447–69,798). Then, each sample were rarified to 24,447 sequences for the evaluation of gut microbiota composition and diversity. A total of 11,151 unique OTUs were detected using UCLSUT clustering. Across all samples, the gut microbiota of Tibetan herdsmen was dominated by Bacteroidetes (54.7%), Firmicutes (37.3%), Tenericutes (3.0%), Proteobacteria (2.4%), and Actinobacteria (2.1%). Other rare phyla (mean relative abundance <1%) included Cyanobacteria, Verrucomicrobia, Fusobacteria, and Lentisphaerae. The gut microbiota composition of each host individual is shown in **Figure 1**.

At genus level, we screened 17 indicative bacterial genera (mean relative abundance > 0.1%) that could be distinguishable among the three groups. Only the significant indicator values that were both significant (*P* < 0.05) and >0.4 were considered (**Table 2**). The most abundant genus in the TH or SUH was *Prevotella*, with an average abundance of 45.86 and 36.14%, respectively. However, the UH was enriched by *Bacteroides* (19.48%). Interestingly, the relative abundance of the dominant *Prevotella* gradually decreased with the degree of urbanization (from TH to SUH to UH). Meanwhile, some bacterial genera, such as *Bacteroides, Faecalibacterium, Blautia, Collinsella, Ruminococcus, Coprococcus*, and *Dorea*, increased with the degree of urbanization.

**Table 2:** The 17 indicative bacterial genera (mean relative abundance > 0.1%) among groups, with their indicator values and significance.

Genera (%)	TH	SUH	UH	Indicator value	FDR-q
*Prevotella*	45.86	36.14	18.59	0.40	<0.01
*Bacteroides*	2.86	11.05	19.48	0.40	<0.01
*Faecalibacterium*	1.49	4.45	6.56	0.40	<0.01
*Oscillospira*	4.86	1.36	2.05	0.40	<0.01
*Roseburia*	1.24	3.21	2.94	0.40	<0.01
*Parabacteroides*	0.57	4.70	0.75	0.40	<0.01
*Catenibacterium*	1.37	3.46	1.03	0.42	0.02
*Blautia*	0.41	0.92	2.61	0.40	<0.01
*Collinsella*	0.18	0.72	2.93	0.40	<0.01
*Ruminococcus*	0.40	1.05	2.36	0.40	<0.01
*Phascolarctobacterium*	1.01	1.49	1.15	0.41	<0.01
*Coprococcus*	0.41	0.60	1.83	0.40	<0.01
*Eubacterium*	0.76	0.84	0.55	0.40	<0.01
*Dialister*	1.18	0.14	0.66	0.49	<0.01
*Sutterella*	0.40	0.56	0.29	0.41	<0.01
*Dorea*	0.10	0.25	0.82	0.40	<0.01
*Odoribacter*	0.09	0.25	0.15	0.44	<0.01

*Only the indicator values that were both significant (P < 0.05) and >0.4 were considered*.

### Variations of Gut Community Diversity With Urbanization

**FIGURE 2:**
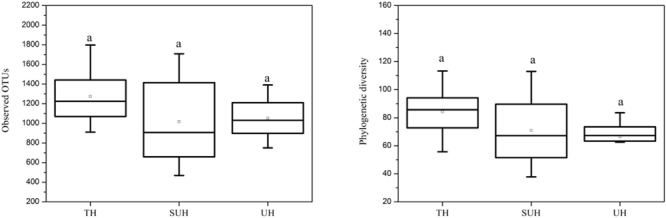
The comparison of alpha diversity indices (observed OTUs and phylogenetic diversity) among three different groups. Significant differences are indicated by different letters. There are no significant differences between any two groups.

**FIGURE 3:**
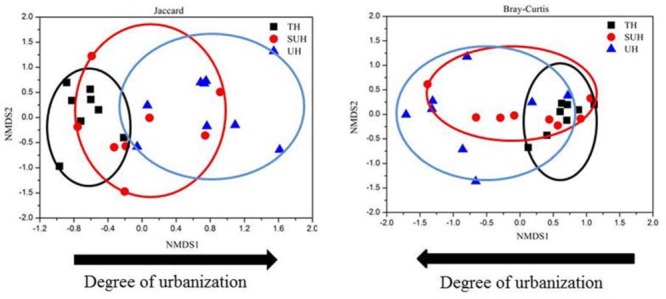
Non-metric multidimensional scaling (NMDS) plots derived from Jaccard and Bray–Curtis distances between samples.

The rarefaction curves almost reached plateau (Supplementary Figure S1), indicating that our sequencing depth was sufficient even addition sequencing may acquire some rare bacterial species. For alpha diversity, the observed OTUs and phylogenetic diversity of gut microbiota showed no significant changes with degree of urbanization (one-way ANOVA, *P* > 0.05, **Figure 2**). For beta diversity, NMDS ordination plots revealed clear differences in the community composition and structure among the three groups (**Figure 3**). We found that lifestyles (or degree of urbanization) significantly influence the community composition (*R*^2^ = 0.204, *P* < 0.001) and structure (*R*^2^ = 0.308, *P* < 0.001) based on the PERMANOVA analysis (Supplementary Table S1). To ensure the reliability of our results, ANOSIM analysis was also used to evaluate the differences of gut microbiota among three groups. Similarly, the gut community composition (*r* = 0.402, *P* < 0.001) and structure (*r* = 0.266, *P* = 0.006) were also impacted by lifestyles (Supplementary Table S1). However, the community composition and structure were not impacted by age, gender, height or sex (*P* > 0.05). We also compared the comparison of gut microbiota between any two groups. The gut community and structure showed significant differences between any two groups based on Jaccard distance. For Bray–Curtis distance, only the group TH and UH showed significant differences in gut microbiota.

**FIGURE 4:**
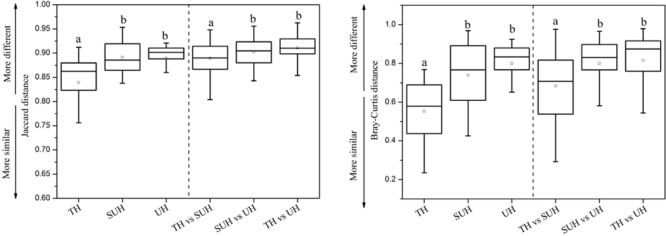
Intra- and inter-group variations of the gut microbiota of the 24 herdsmen that were sampled in all three groups. Significant differences are indicated by different letters.

The inter- and intra-group dissimilarities in gut microbiota composition were calculated based on the Jaccard and Bray–Curtis distance (**Figure 4**). The intra-group variation in the group TH was lower than those in other groups. However, the group UH showed a greater variation of gut microbiota than other groups. For inter-group variation between groups, we found that the group TH was more similar to the group SUH than UH.

### Shifts of Gene Functions With Urbanization

The NMDS plots of the Bray–Curtis dissimilarity based on predicted gene functions at level 3 showed that the overall urban lifestyle showed no significant effects in the functional profiles (PERMANOVA, *R*^2^ = 0.09, *P* = 0.421; ANOSIM, *r* = 0.011, *P* = 0.372, Supplementary Figure S2). When we compared the functional profiles between any two groups, we found that only the TH and UH had slightly different functional profiles (PERMANOVA, *R*^2^ = 0.141, *P* = 0.094; ANOSIM, *r* = 0.149, *P* = 0.074). Thus we compared the differences between the group TH and UH. The main functions associated with metabolism, such as carbohydrate metabolism, lipid metabolism, metabolism of cofactors and vitamins, biosynthesis of other secondary metabolites, xenobiotics biodegradation and metabolism, showed significant differences between TH and UH (FDR-q < 0.05, Supplementary Table S2).

### Changes of Network Topological Features With Urbanization

**FIGURE 5:**
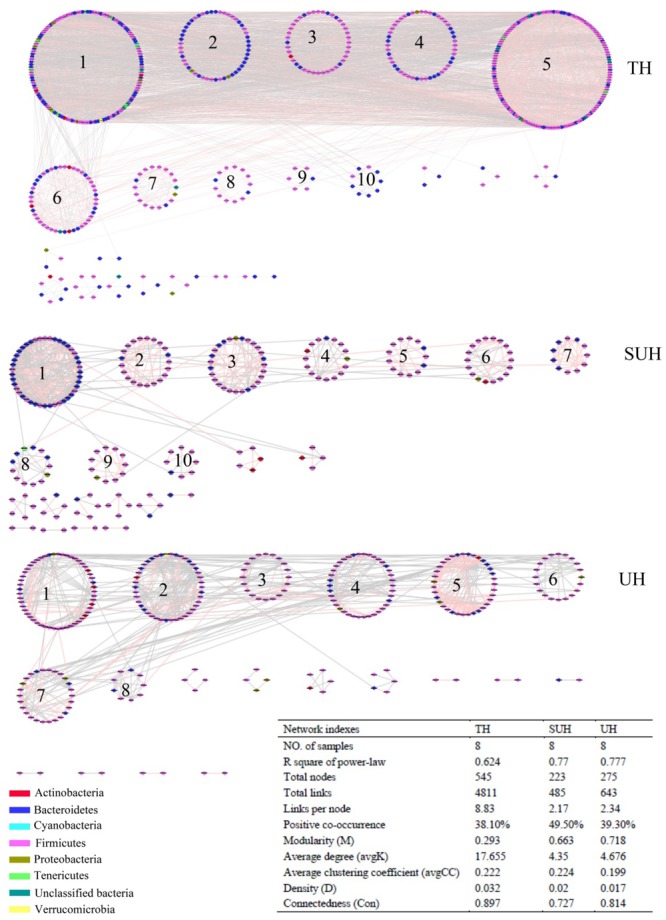
Comparison of gut bacterial networks among three groups. Highly positive correlation is marked by red color and negative correlation by gray color. Lower average degree (avgK) and density (D) indicates a simpler network. A lower average clustering coefficient (avgCC) means that the bacterial network is mainly composed of relatively isolated nodes. The RMT cutoff value for each network is 0.89.

**FIGURE 6:**
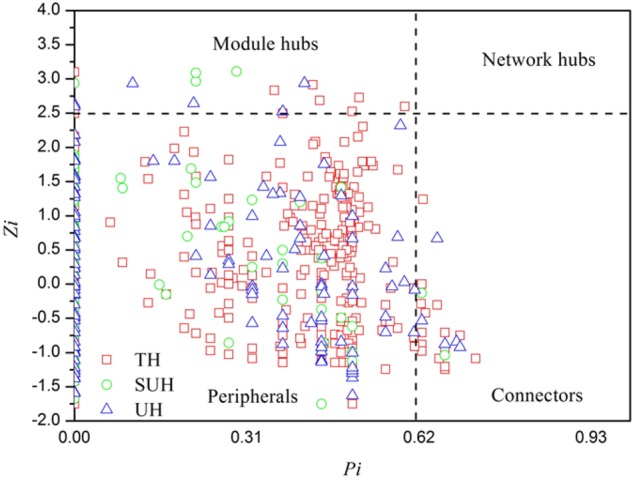
Z-P plot showing the distribution of bacterial OTUs based on their topological features. The topological role of each OTU was determined according to the scatter plot of within-module (Zi) and among-module (Pi) values. Those OTUs that were classified as module hubs, network hubs and connectors were considered as keystone species in bacterial networks.

To understand the co-occurrence patterns of gut microbiota among different lifestyles, we evaluated and compared the interspecies interaction in each group. The OTU table was split into three datasets, namely TH, SUH, and UH. The OTUs detected in more than 50% samples in each group were selected for correlation calculation, and finally resulting in 545, 223, and 275 OTUs from the group TH, SUH, and UH, respectively (**Figure 5**). In the three networks, the connectivity distribution curves matched with the power law model (*R*^2^ range of 0.624–0.777), indicating that the constructed networks were scale-free. In other words, the degree distribution of our constructed networks follows the power law, at least asymptotically. The average degree (avgK) and density (D) of SUH and UH were lower than TH, indicating the TH harbored a more complex ecological network (more complex interaction among microbes). However, SUH and UH had higher positive occurrences than TH, suggesting that less cooperative interspecies interaction in the latter. In addition, the connectedness (Con) of TH was higher than SUH and UH, the nodes in the bacterial network from TH were better connected. Moreover, we also found that modularity increased with degree of urbanization (from TH to SUH to UH), indicating that the niches in the UH may be occupied by more different patches of co-existing functional units. Notably, the topological roles of bacterial taxa were distinct among groups (**Figure 6**). For instance, there were more keystone species in the network of TH than those in other groups. Taken together, the urbanization of Tibetan herdsmen seemed to weaken interspecies interactions, and resulted in the shifts of keystone bacterial species in networks.

### Dynamics of Ecological Processes With Urbanization

**FIGURE 7:**
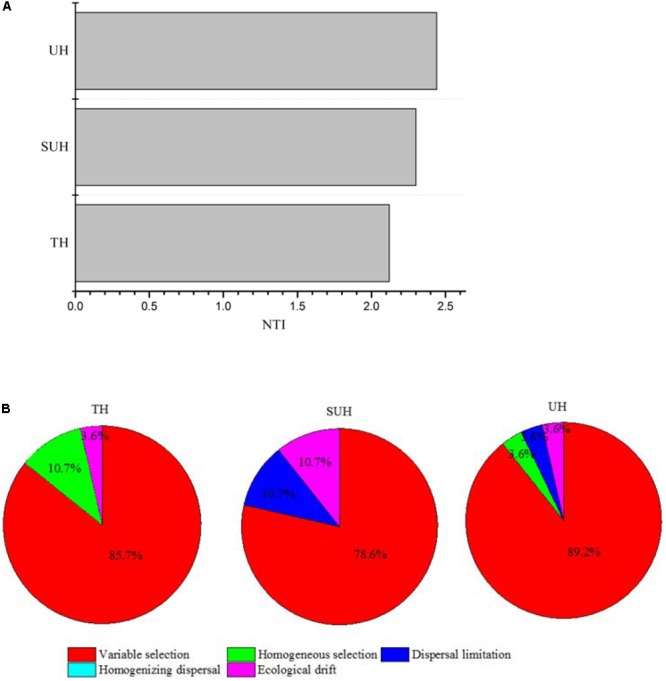
Nearest Taxon Index values and ecological processes of gut microbiota for herdsmen from three different lifestyles. **(A)** The mean NTI values were calculated in different sample types. **(B)** Summary of the relative contributions of the ecological processes that determine community assembly across groups.

The PERMDISP results were significantly different from the null random expectation for each group based on the Jaccard and Bray–Curtis distances (*P* < 0.01 in all cases, **Table 3**), indicating that the gut microbiota of those herdsmen were mainly governed by deterministic rather than stochastic processes based on taxonomy-based metrics. In addition, we compared the mean NTI for each group (**Figure 7A**) via the phylogenetic analysis. Also, the NTI values for each group were significantly greater than zero (*P* < 0.001), indicating that the compositions of gut microbiota for each group were primarily shaped by phylogenetic clustering (or environmental filtering) based on phylogenetic analysis. The absolute magnitude of NTI values increased with degree of urbanization (from TH to SUH to UH), indicating that the relative importance of environmental filtering increases with degree of urbanization in the Tibetan herdsmen.

**Table 3:** Significance test of the differences of centroids between the gut microbial communities and null model simulations across different groups.

	Actual centroid	Null centroid	*F*	*P*
**Jaccard distance**				
TH	0.555	0.487	15.732	**0.0014**
SUH	0.589	0.504	65.132	**1.24E-06**
UH	0.589	0.489	85.454	**2.45E-7**
**Bray–Curtis distance**				
TH	0.362	0.638	28.058	**0.00011**
SUH	0.488	0.642	9.695	**0.0076**
UH	0.532	0.639	11.852	**0.004**

*Permutational analysis of multivariate dispersions (PERMDISP) was used. P-values < 0.01 were in bold.*

In addition, we determined which ecological processes governed the gut microbial communities of those Tibetan herdsmen. The process variable selection, which causes differences in community composition under different selective environments among local scales, was mainly responsible for the assembly of gut microbial communities in Tibetan herdsmen (78.6–89.2%, **Figure 7B**), while dispersal limitation, which causes divergence in community composition due to limited exchange of microbes, only contributed 0–10.7% to the community assembly.

## Discussion

Urbanization has led to lifestyle changes of Tibetan herdsmen in the Qinghai-Tibet Plateau. However, knowledge regarding how the gut microbiota adapts to the changing lifestyles remains insufficient. Here, we explored the diversity, interaction and assembly of gut microbiota in the Tibetan herdsmen with different levels of urbanization. We found that gut microbiota is influenced by lifestyles, and has a certain adaptation in response to urban lifestyles.

### Diversity of Gut Microbiota Reflects the Degree of Urbanization

Recent studies suggest that urbanization was related to a loss of gut microbial diversity in humans (Winglee et al., 2017). However, our data showed that the microbial diversity of Tibetan herdsmen had no significant differences among different levels of urbanization. This result may be attributed to the following reasons: (i) This study represents an early stage of urbanization (only several years of ecological migration), the loss of gut microbial diversity in response to diet shifts was more pronounced with more new generations (Sonnenburg et al., 2016); or (ii) our results was based on the limited sample size (only eight individuals in each group), which may influence the diversity comparison between Tibetan herdsmen. Although there are no statistic significances, we observed a decreased trend of alpha diversity in semi-urban and urban herdsmen compared with traditional herdsmen (**Figure 2**). Thus we speculate that if we continue to sample these populations in greater sample size over generations, the differences of gut microbial diversity will become pronounced.

Our results showed that the gut communities were not impacted by age, gender, height or sex, while urbanization lifestyles influenced the community composition and structure. This finding indicated that the variations in microbial lineages and abundance were primarily impacted by urbanization. These results were largely consistent with previous studies, which found that urbanization/industrialized humans had significant different beta diversity of gut microbiota compared with those humans in traditional rural societies (Schnorr et al., 2014; Martinez et al., 2015). In our study, the dietary configurations have undergone greater shifts from traditional herdsmen (TH) to semi-urban herdsmen (SUH) to urban herdsmen (UH). For instance, the main food in the TH was Zanba (high-fiber food), while the main diet composition in the UH was noodles and rice, which has a low fiber content compared to Zanba. Thus, the shifts of diet configurations may shape the beta diversity of gut microbiota. High-fiber diets in traditional herdsmen may also influence specific gut microbiota taxa. For example, the genus *Prevotella* in traditional herdsmen showed a higher abundance than semi-urban and urban herdsmen. High abundance of *Prevotella* was often associated with the carbohydrate-based or high-fiber diet, a typical characteristic of traditional rural populations, rather than urbanization diet rich in animal protein and high-fat food (Wu et al., 2011). In contrast, the relative abundance of *Bacteroides, Faecalibacterium, Blautia*, and *Ruminococcus* increased with the degree of urbanization, the results were consistent with previous reports, which found that westernized or industrialization societies harbored more these bacterial genera (Yatsunenko et al., 2012; Schnorr et al., 2014). We found that the urbanization herdsmen harbored similar compositional patterns of abundant microbes with those westernized societies. Possibly because urban lifestyle has resulted in similar gut microbiota compositions in Westernized and Chinese populations.

Significant differences were found in the gut microbiota between any two groups based on the Jaccard dissimilarity metrics, whereas only the microbiota of TH showed significant differences with that of UH based on the Bray–Curtis dissimilarity metrics. These results indicated that species loss or replacement (changes in species taxa) plays a more important role than species sorting (changes in abundance) from traditional to urban lifestyles. Thus, lifestyle changes should modulate the shifts of species taxa prior to species abundance. Further studies should demonstrate this inference.

We found that gut microbiota composition was more similar among traditional herdsmen than among semi-urban or urban herdsmen (**Figure 4**). A possible explanation is that the former has a narrower diet profile than latter because of limited food sources in the traditional herdsmen. Thus less diverse food profile may cause more similar gut microbiota in the traditional herdsmen. Our previous study has shown that the composition and structure of diet were associated with those of gut microbiota (Li et al., 2016a). In this study, the food profile of traditional herdsmen was more similar to that of semi-urban herdsmen rather than urban herdsmen, thus the gut microbiota of traditional herdsmen was more similar to that of semi-urban herdsmen (**Figure 4**).

Relative to divergent gut microbiota composition across groups, we found that urbanization lifestyle had no significant impacts in shaping the overall functions, indicating the functional shifts was probably relatively slower than compositional changes. However, the TH and UH still showed slight differences in the functional profiles. In particular, the functions associated with carbohydrate metabolism, lipid metabolism and metabolism of cofactors and vitamins showed significant differences between the two groups. These results were partly in accordance with previous studies on the gut microbiome of traditional hunter-gatherers, which live on high fiber food and were enriched in carbohydrate metabolism, lipid metabolism, and vitamin metabolism (Rampelli et al., 2015). The functional differences may be related to the dietary differences between traditional and urban lifestyles, as the urban herdsmen had relatively low-fiber main food (rice and noodle) and more diverse meat, while the traditional herdsmen had high-fiber zanba and less diverse meat.

### Network Interaction of Gut Microbiota Is Associated With Urbanization

It is generally accepted that microbial interaction is able to mediate the microbial function (Ramayo-Caldas et al., 2016). We found that the gut microbial interaction of traditional herdsmen was more complex and better connected (**Figure 5**). One recent network analysis showed that obese children had a higher density of gut microbiota compared with normal-weight children, which was consistent with an increased fermentation capacity for food (Riva et al., 2016). Thus more complex microbial interactions in the traditional herdsmen may be beneficial to the decomposition of indigestible high-fiber food by improving the fermentation capacity. In addition, the gut microbiota of traditional herdsmen exhibited a lower percentage of positive co-occurrence, indicating that the microbial community in traditional herdsmen included more percentage of competition rather cooperative interactions. Strong competition in a diversity community is likely to favor allelopathic species (Inglis et al., 2009; Jousset et al., 2011). Increased allelopathic interaction may possibly improve the resident microbial community barrier again exotic pathogen invasion (Jousset et al., 2011). Under this situation, urban populations were likely more prone to pathogen invasion. Indeed, one recent report has showed that urbanization has led to a loss of potentially beneficial bacteria and an increase of potential pathogenic bacteria or genes (Winglee et al., 2017). Future studies should focus on the relationship between gut microbial interactions, pathogen invasion and diseases.

In particular, we found that the network modularity of traditional herdsmen increased with urbanization (**Figure 5**). High modularity value indicates a high level of niche differentiation. It has been shown that increased niche differentiation may result in weaker microbial interactions in the soil (Faust and Raes, 2012), as was supported by our results, which showed that urban herdsmen harbored less links and density. In our study, the increased niche differentiation in urban populations may be associated with more diverse food, in order to improve the digestion of different diets. Our previous study has also demonstrated that increased network modularity may improve the functional redundancy and efficiency of anaerobic digestion systems (Lin et al., 2017). In addition, we found that the network of TH group harbored more putative keystone species than SUH or UH, implying that the loss of one keystone species may possibly not influence the community function in traditional herdsmen. However, our network metrics were dependent on limited sample size, sequencing depth and data processing methods, thereby possibly producing some deviations for topological features. For example, to ensure correlation reliability and improve statistical confidence, only those OTUs that were present in at least 50% samples were used for network construction, however, this processing may have influenced the topological features of bacterial networks.

### Variable Selection Plays a Major Role in the Assembly of Gut Microbial Communities

Our results showed that the assembly of gut microbiota was mainly governed by deterministic processes, indicating that environmental filtering or host-specific selection may lead to assembly of microbial communities in Tibetan herdsmen. Some host (e.g., host genetics or immunity) or environmental (e.g., diet) factors may enrich or maintain those bacteria that adapted for gut environment. Null model (Jaccard or Bray–Curtis distance) and phylogenetic-based MNTD analysis (NTI) suggested that gut microbial lineages, abundances and phylogenetic distance was likely to be influenced by those deterministic factors. Our results were consistent with the previous studies on the gut microbiota of aquatic animals (e.g., fish and shrimp), which found that gut microbiota was primarily shaped by deterministic processes (Yan et al., 2016; Xiong et al., 2017). Thus, deterministic processes should play a dominant role in the gut community assembly of mammalian and fish due to the filtering of digestive tract (e.g., gut environment) and selection of host specificity (e.g., body temperature). Notably, we found that the relative contributions of environmental filtering increase with urbanization. Although in traditional populations diet seems to be much more restricted, it would seem that stochastic factors (e.g., stochastic dispersal) may play more important roles in structuring the gut microbiota, thereby resulting in a smaller proportion of environmental filtering for community assembly in TH.

The traditional herdsmen are nomadic and live in tents, thus their houses are always mobile. The rates of inter-individual gut microbial dispersal (which relates to horizontal transmission) is likely to very low compared with urban populations. This hypothesis is concordant with our results, as we did not detect the relative contributions of dispersal in the traditional herdsmen. However, our results did not imply that there was no physical possibility of microbial dispersion, just that microbial dispersion patterns were not reflected in community structure due to the stronger role of selection. In contrast, semi-urban and urban populations have more gathered houses, which may result in more frequent horizontal transmission between individuals. This lifestyle of urbanized herdsmen may increase the rates of microbial transmission among individuals, although not sufficiently to result in homogenizing effects across the population, therefore resulting in a detectable degree of dispersal limitation contributing to the overall beta diversity. Indeed we found that the relative contributions of dispersal limitation in the semi-urban and urban populations were 10.7 and 3.6%, respectively. As the population migration occurred in the current generation starting in 2004, the analyzed fecal samples of SUH and UH correspond to individuals who were relatively recently relocated from their traditional environment toward more urban environments. Further studies should estimate how migration influences microbial community assembly in the gut during urbanization in the longer term.

Variable selection plays a dominant role in the assembly of gut microbiota in each group. The contribution differences of variable selection in the three groups may be derived from different environmental pressures, such as nomadic lifestyle, geography, host genetics and diet. Previous reports showed that there were no major differences of human microbiota between different industrialized countries across nations (Arumugam et al., 2014), indicating that genetic and geographical factors exert minor effects. Thus, other factors, such as diet, nomadic lifestyle and population density, may shape the ecological processes. There was a stronger signal for variable selection in the urban herdsmen compared with the traditional herdsmen. One possible cause for increased beta diversity in urban populations might be the larger variation in diet among urbanized herdsmen. This would be in line with the increase in variable selection in UH. Also, selection may limit the colonization of foreign species and filter those bacteria that are not adapted for the ecosystem, thus decreasing the alpha diversity (Costello et al., 2012). Our results support these speculations because urban population, which had high variable selection, harbored lower alpha diversity and higher beta diversity compared with nomadic, traditional herdsmen. However, the accurate assessment regarding the effects of environmental factors on the ecological processes in traditional and urban humans needs further study.

## Conclusion

We found that the composition and structure of the gut microbiota are associated with urbanization. Fiber-degrading bacteria (e.g., *Prevotella*) are abundant in the traditional Tibetan herdsmen, whereas those bacteria (e.g., *Bacteroides*) associated with diets rich in animal protein are dominant in the urbanized herdsmen. In comparison with traditional herdsmen, the microbial interaction of urbanized herdsmen becomes weakened. However, there was a stronger signal of variable selection in the urban than traditional herdsmen, implying that more different selective pressures cause divergent gut community compositions in response to urban lifestyles. These findings indicate that urban lifestyles not only mediate the composition and structure of human gut microbiota, but also influence the microbial interaction and community assembly patterns. Further studies should evaluate the effects of gut microbiota on human health during urbanization.

## Author Contributions

HL and JQ conceived the experimental design. HL, TL, and JQ finished the field experiments. HL finished the data analysis and wrote the original manuscript. All authors revised the manuscript.

## Conflict of Interest Statement

The authors declare that the research was conducted in the absence of any commercial or financial relationships that could be construed as a potential conflict of interest.

## Footnotes

1 http://www.ebi.ac.uk/ena/data/view/PRJEB23227

## References

[B1] Arumugam M. Raes J. Pelletier E. Le Paslier D. Yamada T. Mende D. R. (2014). Enterotypes of the human gut microbiome. *Nature* 473 174–180. 10.1038/nature09944 21508958 PMC3728647

[B2] Caporaso J. G. Kuczynski J. Stombaugh J. Bittinger K. Bushman F. D. Costello E. K. (2010). QIIME allows analysis of high-throughput community sequencing data. *Nat. Methods* 7 335–336. 10.1038/nmeth.f.303 20383131 PMC3156573

[B3] Carrillo-Larco R. M. Bernabe-Ortiz A. Pillay T. D. Gilman R. H. Sanchez J. F. Poterico J. A. (2016). Obesity risk in rural, urban and rural-to-urban migrants: prospective results of the PERU MIGRANT study. *Int. J. Obes.* 40 181–185. 10.1038/ijo.2015.140 26228458 PMC4677453

[B4] Costello E. K. Stagaman K. Dethlefsen L. Bohannan B. J. Relman D. A. (2012). The application of ecological theory toward an understanding of the human microbiome. *Science* 336 1255–1262. 10.1126/science.1224203 22674335 PMC4208626

[B5] Deng Y. Jiang Y. H. Yang Y. He Z. Luo F. Zhou J. (2012). Molecular ecological network analyses. *BMC Bioinformatics* 13:113. 10.1186/1471-2105-13-113 22646978 PMC3428680

[B6] DeSantis T. Hugenholtz P. Larsen N. Rojas M. Brodie E. Keller K. (2006). Greengenes, a chimera-checked 16S rRNA gene database and workbench compatible with ARB. *Appl. Environ. Microbiol.* 72 5069–5072. 10.1128/AEM.03006-05 16820507 PMC1489311

[B7] Dill-McFarland K. A. Weimer P. J. Pauli J. N. Peery M. Z. Suen G. (2015). Diet specialization selects for an unusual and simplified gut microbiota in two- and three-toed sloths. *Environ. Microbiol.* 18 1391–1402. 10.1111/1462-2920.13022 26271635

[B8] Dufrêne M. Legendre P. (1997). Species assemblages and indicator species: the need for a flexible asymmetrical approach. *Ecol. Monogr.* 67 345–366. 10.2307/2963459

[B9] Edgar R. C. (2010). Search and clustering orders of magnitude faster than BLAST. *Bioinformatics* 26 2460–2461. 10.1093/bioinformatics/btq461 20709691

[B10] Faust K. Raes J. (2012). Microbial interactions: from networks to models. *Nat. Rev. Microbiol.* 10 538–550. 10.1038/nrmicro2832 22796884

[B11] Gomez A. Petrzelkova K. J. Burns M. B. Yeoman C. J. Amato K. R. Vlckova K. (2016). Gut microbiome of coexisting BaAka pygmies and bantu reflects gradients of traditional subsistence patterns. *Cell Rep.* 14 2142–2153. 10.1016/j.celrep.2016.02.013 26923597

[B12] Inglis R. Gardner A. Cornelis P. Buckling A. (2009). Spite and virulence in the bacterium *Pseudomonas Aeruginosa*. *Proc. Natl. Acad. Sci. U.S.A.* 106 5703–5707. 10.1073/pnas.0810850106 19321425 PMC2667014

[B13] Jia Y. (2016). Review of benefit evaluation research on ecological migration in China. *Resour. Sci.* 38 87–92.

[B14] Jousset A. Schulz W. Scheu S. Eisenhauer N. (2011). Intraspecific genotypic richness and relatedness predict the invasibility of microbial communities. *ISME J.* 5 1108–1114. 10.1038/ismej.2011.9 21346790 PMC3146292

[B15] Kara E. L. Hanson P. C. Hu Y. H. Winslow L. McMahon K. D. (2012). A decade of seasonal dynamics and co-occurrences within freshwater bacterioplankton communities from eutrophic Lake Mendota. WI, USA. *ISME J.* 7 680–684. 10.1038/ismej.2012.118 23051691 PMC3578560

[B16] Kembel S. W. Cowan P. D. Helmus M. R. Cornwell W. K. Morlon H. Ackerly D. D. (2010). Picante: R tools for integrating phylogenies and ecology. *Bioinformatics* 26 1463–1464. 10.1093/bioinformatics/btq166 20395285

[B17] Langille M. G. Zaneveld J. Caporaso J. G. McDonald D. Knights D. Reyes J. A. (2013). Predictive functional profiling of microbial communities using 16S rRNA marker gene sequences. *Nat. Biotech.* 31 814–821. 10.1038/nbt.2676 23975157 PMC3819121

[B18] Li H. Li T. Beasley D. E. Hedenec P. Xiao Z. Zhang S. (2016a). Diet diversity is associated with beta but not alpha diversity of pika gut microbiota. *Front. Microbiol.* 7:1169 10.3389/fmicb.2016.01169 PMC4961685 27512391

[B19] Li H. Li T. Tu B. Kou Y. Li X. (2017). Host species shapes the co-occurrence patterns rather than diversity of stomach bacterial communities in pikas. *Appl. Microbiol. Biotechnol.* 101 5519–5529. 10.1007/s00253-017-8254-0 28365795

[B20] Li H. Li T. Yao M. Li J. Zhang S. Wirth S. (2016b). Pika gut may select for rare but diverse environmental bacteria. *Front. Microbiol.* 7:1269. 10.3389/fmicb.2016.01269 27582734 PMC4987353

[B21] Li H. Qu J. Li T. Li J. Lin Q. Li X. (2016c). Pika population density is associated with composition and diversity of gut microbiota. *Front. Microbiol.* 7:758. 10.3389/fmicb.2016.00758 27242770 PMC4870984

[B22] Lin Q. De Vrieze J. Li C. Li J. Li J. Yao M. (2017). Temperature regulates deterministic processes and the succession of microbial interactions in anaerobic digestion process. *Water Res.* 123 134–143. 10.1016/j.watres.2017.06.051 28662395

[B23] Magoc T. Salzberg S. L. (2011). FLASH: fast length adjustment of short reads to improve genome assemblies. *Bioinformatics* 27 2957–2963. 10.1093/bioinformatics/btr507 21903629 PMC3198573

[B24] Martinez I. Stegen J. C. Maldonado-Gomez M. X. Eren A. M. Siba P. M. Greenhill A. R. (2015). The gut microbiota of rural papua new guineans: composition, diversity patterns, and ecological processes. *Cell Rep.* 11 527–538. 10.1016/j.celrep.2015.03.049 25892234

[B25] McArdle B. H. Anderson M. J. (2001). Fitting multivariate models to community data: a comment on distance-based redundancy analysis. *Ecology* 82 290–297. 10.1890/0012-9658(2001)082[0290:FMMTCD]2.0.CO;2

[B26] Meyerhof M. S. Wilson J. M. Dawson M. N. Michael Beman J. (2016). Microbial community diversity, structure and assembly across oxygen gradients in meromictic marine lakes, Palau. *Environ. Microbiol.* 18 4907–4919. 10.1111/1462-2920.13416 27312889

[B27] Obregon-Tito A. J. Tito R. Y. Metcalf J. Sankaranarayanan K. Clemente J. C. Ursell L. K. (2015). Subsistence strategies in traditional societies distinguish gut microbiomes. *Nat. Commun.* 6:6505. 10.1038/ncomms7505 25807110 PMC4386023

[B28] O’Keefe S. J. Li J. V. Lahti L. Ou J. Carbonero F. Mohammed K. (2015). Fat, fibre and cancer risk in African Americans and rural Africans. *Nat. Commun.* 6:6342. 10.1038/ncomms7342 25919227 PMC4415091

[B29] Purves D. W. Turnbull L. A. (2010). Different but equal: the implausible assumption at the heart of neutral theory. *J. Anim. Ecol.* 79 1215–1225. 10.1038/ncomms7342 20726922 PMC3025117

[B30] Ramayo-Caldas Y. Mach N. Lepage P. Levenez F. Denis C. Lemonnier G. (2016). Phylogenetic network analysis applied to pig gut microbiota identifies an ecosystem structure linked with growth traits. *ISME J.* 10 2973–2977. 10.1038/ismej.2016.77 27177190 PMC5148198

[B31] Rampelli S. Schnorr S. L. Consolandi C. Turroni S. Severgnini M. Peano C. (2015). Metagenome sequencing of the Hadza hunter-gatherer gut Microbiota. *Curr. Biol.* 25 1682–1693. 10.1016/j.cub.2015.04.055 25981789

[B32] Riva A. Borgo F. Lassandro C. Verduci E. Morace G. Borghi E. (2016). Pediatric obesity is associated with an altered gut microbiota and discordant shifts in Firmicutes populations. *Environ. Microbiol.* 48:e268. 10.1111/1462-2920.13463 27450202 PMC5516186

[B33] Roberts D. (2007). labdsv: ordination and multivariate analysis for ecology. *R Package Version* 1:3.

[B34] Rosindell J. Hubbell S. P. He F. Harmon L. J. Etienne R. S. (2012). The case for ecological neutral theory. *Trends Ecol. Evol.* 27 203–208. 10.1016/j.tree.2012.01.004 22341498

[B35] Saito R. Smoot M. Ono K. Ruscheinski J. Wang P. Lotia S. (2012). A travel guide to cytoscape plugins. *Nat. Methods* 9 1069–1076. 10.1038/nmeth.2212 23132118 PMC3649846

[B36] Schnorr S. L. Candela M. Rampelli S. Centanni M. Consolandi C. Basaglia G. (2014). Gut microbiome of the Hadza hunter-gatherers. *Nat. Commun.* 5:3654. 10.1038/ncomms4654 24736369 PMC3996546

[B37] Song H.-N. Go S.-I. Lee W. S. Kim Y. Choi H. J. Lee U. S. (2016). Population-based regional cancer incidence in Korea: comparison between urban and rural areas. *Cancer Res. Treat.* 48 789–797. 10.4143/crt.2015.062 26194369 PMC4843717

[B38] Sonnenburg E. D. Smits S. A. Tikhonov M. Higginbottom S. K. Wingreen N. S. Sonnenburg J. L. (2016). Diet-induced extinctions in the gut microbiota compound over generations. *Nature* 529 212–215. 10.1038/nature16504 26762459 PMC4850918

[B39] Stegen J. C. Lin X. Fredrickson J. K. Chen X. Kennedy D. W. Murray C. J. (2013). Quantifying community assembly processes and identifying features that impose them. *ISME J.* 7 2069–2079. 10.1038/ismej.2013.93 23739053 PMC3806266

[B40] Stegen J. C. Lin X. Fredrickson J. K. Konopka A. E. (2015). Estimating and mapping ecological processes influencing microbial community assembly. *Front. Microbiol.* 6:370. 10.3389/fmicb.2015.00370 25983725 PMC4416444

[B41] Stegen J. C. Lin X. Konopka A. E. Fredrickson J. K. (2012). Stochastic and deterministic assembly processes in subsurface microbial communities. *ISME J.* 6 1653–1664. 10.1038/ismej.2012.22 22456445 PMC3498916

[B42] Tamaki H. Wright C. Li X. Lin Q. Hwang C. Wang S. (2011). Analysis of 16S rRNA amplicon sequencing options on the Roche/454 next-generation titanium sequencing platform. *PLoS One* 6:e25263. 10.1371/journal.pone.0025263 21966473 PMC3179495

[B43] Vellend M. (2010). Conceptual synthesis in community ecology. *Q. Rev. Biol.* 85 183–206. 10.1086/652373 20565040

[B44] Vellend M. Srivastava D. S. Anderson K. M. Brown C. D. Jankowski J. E. Kleynhans E. J. (2014). Assessing the relative importance of neutral stochasticity in ecological communities. *Oikos* 123 1420–1430. 10.1111/oik.01493

[B45] Wang Q. Garrity G. M. Tiedje J. M. Cole J. R. (2007). Naive Bayesian classifier for rapid assignment of rRNA sequences into the new bacterial taxonomy. *Appl. Environ. Microbiol.* 73 5261–5267. 10.1128/AEM.00062-07 17586664 PMC1950982

[B46] Werner U. Nicholas J. G. (2010). Null model analysis of species associations using abundance data. *Ecology* 91 3384–3397. 10.1890/09-2157.1 21141199

[B47] Winglee K. Howard A. G. Sha W. Gharaibeh R. Z. Liu J. Jin D. (2017). Recent urbanization in China is correlated with a Westernized microbiome encoding increased virulence and antibiotic resistance genes. *Microbiome* 5:121. 10.1186/s40168-017-0338-7 28915922 PMC5603068

[B48] Wu G. D. Chen J. Hoffmann C. Bittinger K. Chen Y. Y. Keilbaugh S. A. (2011). Linking long-term dietary patterns with gut microbial enterotypes. *Science* 334 105–108. 10.1126/science.1208344 21885731 PMC3368382

[B49] Xiong J. Dai W. Zhu J. Liu K. Dong C. Qiu Q. (2017). The underlying ecological processes of gut microbiota among cohabitating retarded, overgrown and normal shrimp. *Microb. Ecol.* 73 988–999. 10.1007/s00248-016-0910-x 27966036

[B50] Yan Q. Li J. Yu Y. Wang J. He Z. Van Nostrand J. D. (2016). Environmental filtering decreases with fish development for the assembly of gut microbiota. *Environ. Microbiol.* 18 4739–4754. 10.1111/1462-2920.13365 27130138

[B51] Yatsunenko T. Rey F. E. Manary M. J. Trehan I. Dominguez-Bello M. G. Contreras M. (2012). Human gut microbiome viewed across age and geography. *Nature* 486 222–227. 10.1038/nature11053 22699611 PMC3376388

[B52] Zhou J. Deng Y. Zhang P. Xue K. Liang Y. Van Nostrand J. D. (2014). Stochasticity, succession, and environmental perturbations in a fluidic ecosystem. *Proc. Natl. Acad. Sci. U.S.A.* 111 E836–E845. 10.1073/pnas.1324044111 24550501 PMC3948316

[B53] Zhou M. Astell-Burt T. Yin P. Feng X. Page A. Liu Y. (2015). Spatiotemporal variation in diabetes mortality in China: multilevel evidence from 2006 and 2012. *BMC Public Health* 15:633. 10.1186/s12889-015-1982-0 26159911 PMC4496807

