# Supplementary material for: Gut Microbiota in Tibetan Herdsmen Reflects the Degree of Urbanization
Source: Front Microbiol. 2018 Jul 31;9:1745. doi: 10.3389/fmicb.2018.01745 (PMC6080570; doi:10.3389/fmicb.2018.01745)
Supplement: Supplementary file 1 [file Table_1.PDF]

Table S1. The comparision of gut microbiomes composition and structure among three groups.

	Jaccard distance				Bray-Curtis distance			
	PERMANOVA		ANOSIM		PERMANOVA		ANOSIM	
	R^2	P	R	P	R^2	P	R	P
Lifestyle	0.204	**<0.001**	0.402	**<0.001**	0.308	**0.011**	0.266	**0.006**
TH vs SUH	0.134	**0.013**	0.312	**0.017**	0.142	0.071	0.089	0.154
SUH vs UH	0.096	**0.029**	0.17	**0.048**	0.139	0.082	0.094	0.139
TH vs UH	0.224	**<0.001**	0.694	**<0.001**	0.425	**<0.001**	0.604	**0.003**
