# Supplementary material for: Gut Microbiota in Tibetan Herdsmen Reflects the Degree of Urbanization
Source: Front Microbiol. 2018 Jul 31;9:1745. doi: 10.3389/fmicb.2018.01745 (PMC6080570; doi:10.3389/fmicb.2018.01745)
Supplement: Supplementary file 2 [file Table_2.PDF]

Table S2. The differences of predicted gene functions at level 3 between the group TH and UH

Function(Level 3)	TH_mean	UH_mean	*P*
Environmental Information Processing; Signaling Molecules and Interaction; Bacterial toxins	0.00087	0.00117	0.0326
Environmental Information Processing; Signaling Molecules and Interaction; Ion channels	0.00007	0.00014	0.0282
Genetic Information Processing; Folding, Sorting and Degradation; Proteasome	0.00055	0.00046	0.0308
Genetic Information Processing; Replication and Repair; Chromosome	0.01851	0.0166	0.0379
Human Diseases; Cancers; Pathways in cancer	0.00058	0.00047	0.0269
Human Diseases; Infectious Diseases; Tuberculosis	0.00173	0.0016	0.03
Metabolism; Biosynthesis of Other Secondary Metabolites; beta-Lactam resistance	0.00011	0.00024	0.0276
Metabolism; Carbohydrate Metabolism; Ascorbate and aldarate metabolism	0.00075	0.00112	0.0318
Metabolism; Carbohydrate Metabolism; Galactose metabolism	0.00704	0.00837	0.0328
Metabolism; Carbohydrate Metabolism; Glyoxylate and dicarboxylate metabolism	0.00418	0.00501	0.0342
Metabolism; Carbohydrate Metabolism; Inositol phosphate metabolism	0.00075	0.0009	0.0478
Metabolism; Lipid Metabolism; Ether lipid metabolism	0.00001	0.00002	0.0461
Metabolism; Lipid Metabolism; Synthesis and degradation of ketone bodies	0.00011	0.00021	0.0401
Metabolism; Metabolism of Cofactors and Vitamins; Porphyrin and chlorophyll metabolism	0.00609	0.00879	0.0302
Metabolism; Metabolism of Cofactors and Vitamins; Vitamin B6 metabolism	0.00263	0.00211	0.0499
Metabolism; Metabolism of Other Amino Acids; D-Alanine metabolism	0.00115	0.00101	0.0306
Metabolism; Xenobiotics Biodegradation and Metabolism; Chlorocyclohexane and chlorobenzene degradation	0.00004	0.00009	0.0296
Metabolism; Xenobiotics Biodegradation and Metabolism; Styrene degradation	0.00007	0.00015	0.0377
Organismal Systems; Endocrine System; Progesterone-mediated oocyte maturation	0.00054	0.00045	0.0388
Organismal Systems; Immune System; Antigen processing and presentation	0.00054	0.00045	0.0311
Organismal Systems; Immune System; NOD-like receptor signaling pathway	0.00055	0.00045	0.0336
Unclassified; Genetic Information Processing; Translation proteins	0.01005	0.00934	0.0322
Unclassified; Metabolism; Energy metabolism	0.01036	0.00908	0.0281
Unclassified; Metabolism; Glycan biosynthesis and metabolism	0.0005	0.00027	0.0284
