# Supplementary figures and images for: Gut Microbiota in Tibetan Herdsmen Reflects the Degree of Urbanization
Source: Front Microbiol. 2018 Jul 31;9:1745. doi: 10.3389/fmicb.2018.01745 (PMC6080570; doi:10.3389/fmicb.2018.01745)

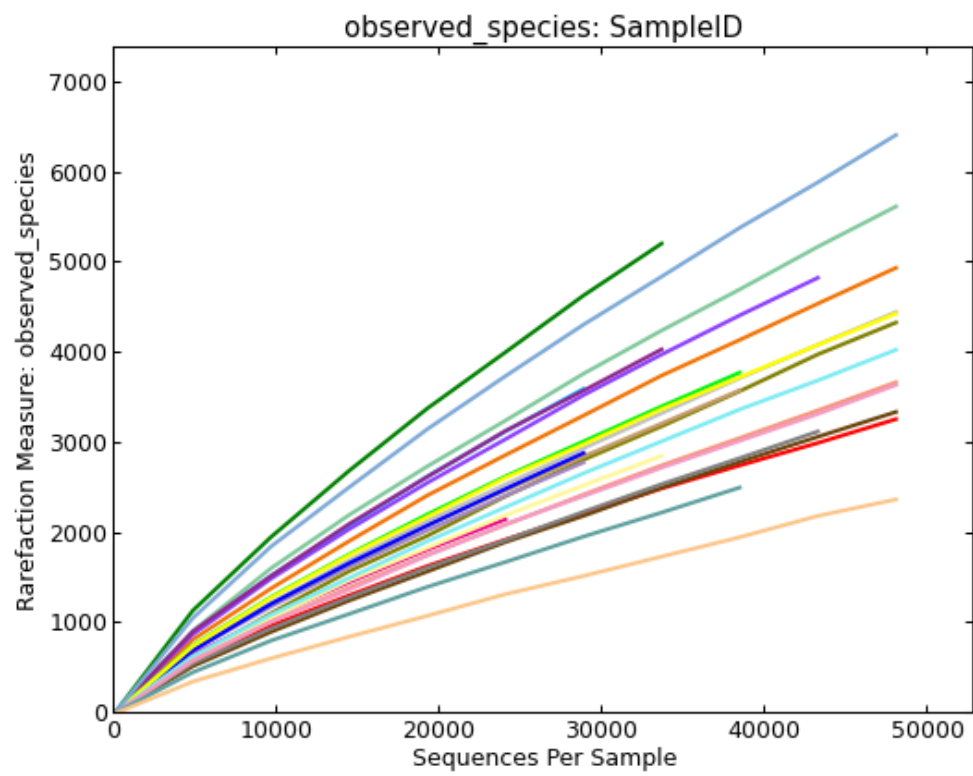

**Figure S1.** The OTU-level rarefaction curves of observed OTUs across all samples.

Supplement: Supplementary file 3 [file Image_1.PDF]
