# Supplementary material for: Gut Microbiota in Tibetan Herdsmen Reflects the Degree of Urbanization
Source: Front Microbiol. 2018 Jul 31;9:1745. doi: 10.3389/fmicb.2018.01745 (PMC6080570; doi:10.3389/fmicb.2018.01745)
Supplement: Supplementary file 4 [file Image_2.PDF]

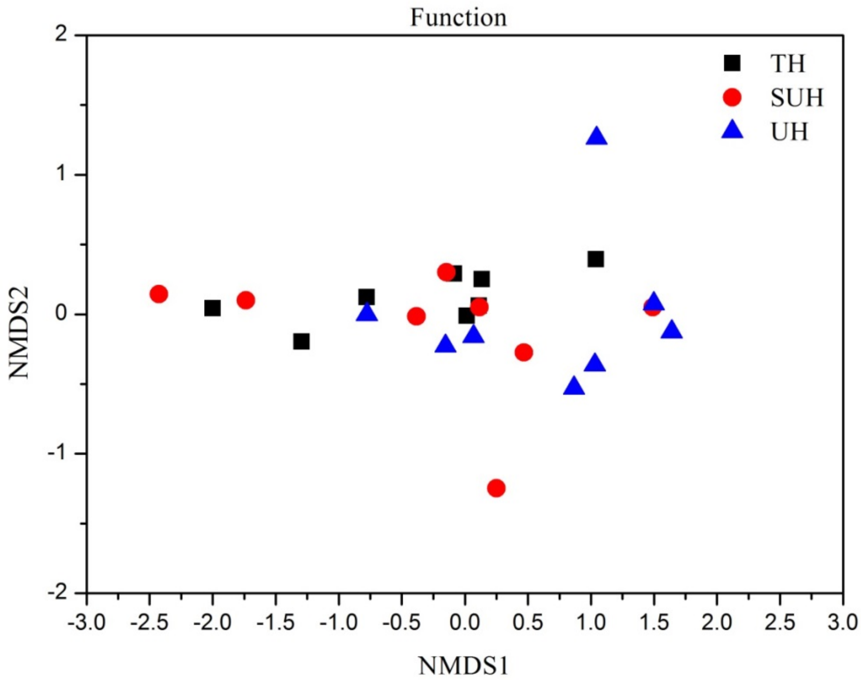

Figure S2. Non-metric multidimensional scaling (NMDS) plots of the Bray-Curtis dissimilarity comparing the predicted gene functions at level 3 among groups
